# Characterization and comparison of the bacterial microbiota in different gastrointestinal tract compartments of Mongolian horses

**DOI:** 10.1002/mbo3.1020

**Published:** 2020-03-09

**Authors:** Shaofeng Su, Yiping Zhao, Zongzheng Liu, Guiqin Liu, Ming Du, Jing Wu, Dongyi Bai, Bei Li, Gerelchimeg Bou, Xinzhuang Zhang, Manglai Dugarjaviin

**Affiliations:** ^1^ College of Animal Science Inner Mongolia Key Laboratory of Equine Genetics, Breeding and Reproduction Scientific Observing and Experimental Station of Equine Genetics, Breeding and Reproduction, Ministry of Agriculture and Rural Affairs Equine Research Centre Inner Mongolia Agricultural University Hohhot China; ^2^ Biotechnology Research Centre Inner Mongolia Academy of Agricultural and Animal Husbandry Sciences Hohhot China; ^3^ Animal Husbandry and Veterinary Research Institute of Qingdao Qingdao China; ^4^ Agricultural College Liaocheng University Liaocheng China

**Keywords:** 16S rRNA V3‐V4, high‐throughput sequencing, intestinal microbiota, microbiome, Mongolian horses

## Abstract

The intestinal microbiota plays an important role in the health and metabolism of the host. Next‐generation sequencing technology has enabled the characterization of the gut microbiota of several animal species. We analyzed the intestinal microbiota in six different parts of the gastrointestinal tracts (GITs) of five Mongolian horses by sequencing the 16S rRNA gene V3‐V4 hypervariable region. All horses were kept in the natural habitat of the Inner Mongolia grassland. Significant differences were observed among the microbiota compositions of the distinct GIT regions. In addition, while the microbial community structures of the small and large intestine were significantly different, those of the cecum and colon were similar. In the foregut, Firmicutes (65%) and Proteobacteria (23%) were the most abundant, while Firmicutes (45%) and Bacteroidetes (42%) were the most common in the hindgut. At the level of family, Ruminococcaceae (*p* = .203), Lachnospiraceae (*p* = .157), Rikenellaceae (*p* = .122), and Prevotellaceae (*p* = .068) were predominant in the hindgut, while the relative abundance of the *Akkermansia* genus (5.7%, *p* = .039) was higher in the ventral colon. In terms of the putative functions, the ratio of microbial abundance in the different parts of the GIT was similar, the result can help characterize the gut microbial structure of different animals.

## Graphical abstract

The microbial communities of the different parts of the Mongolian horse gastrointestinal tracts were significantly different, and there was greater diversity between the lower gut and upper gut. Direct sampling of the different segments of gastrointestinal tracts provided a more complete diagram of the gut microbiota than fecal analysis. The vegetarian diets and adaptability of Mongolian horses were likely related not only to their stable and complicated gastrointestinal microbiota but also to their special herbivorous digestive physiology.

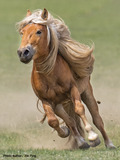

## 1 INTRODUCTION

The horse is a herbivorous nonruminant animal with highly compartmentalized gastrointestinal tract (GIT), which can utilize a variety of plant fibers (Harris et al., 2017; Santos, Rodrigues, Bessa, Ferreira, & Martin‐Rosset, 2011). Each segment of the GIT has an independent ecosystem with unique biotic and abiotic (temperature, water, pH, oxygen, etc.) characteristics. The composition (diversity and structure) and function (metabolic mechanism and end products) of the GIT microbiome are highly significant to animal health and metabolism. In normal circumstances, the gut microbes and host are in the symbiotic and highly dynamic relationship. In horses, for example, 60%–70% energy comes from volatile fatty acids (VFAs) (Argenzio, 1975; Vermorel & MartinRosset, 1997) produced by the cecum and colon microorganisms, 30% of which is produced by the cecum microbiota alone (Glinsky, Smith, Spires, & Davis, 1976). Therefore, the balance and stability of the intestinal microbiota are essential for the health and function of GIT. Several diseases of the GIT are related to change in the composition or function of its microbiota. In addition, metabolic diseases, such as laminitis that can affect the musculoskeletal system, are also related to the intestinal microbiota (Milinovich et al., 2007; Steelman, Chowdhary, Dowd, Suchodolski, & Janecka, 2012).

The Mongolian horse is one of the most ancient grassland horse bred in the world and found in Inner Mongolia, China. At present, studies of the intestinal microorganisms of Mongolian horses have been limited in feces (Zhao et al., 2016). Horse feces can only represent the microbial changes in the distal regions of the posterior intestine (Costa, Silva, et al., 2015; Dougal et al., 2012) rather than the whole gastrointestinal microflora, and this had been demonstrated by studies of human intestinal microflora (Durban et al., 2011; Eckburg et al., 2005). In this study, we analyzed the characterization of the microbial composition of different parts of the Mongolian horse GIT by using the next‐generation sequencing (NGS) firstly.

## 2 MATERIALS AND METHODS

### 2.1 Horses and sample collection

Five healthy Mongolian horses (three males and two females with an average age of 4.4 years ranged from 3 to 6 years and weight of 292.8 ± 11.9 Kg) grazed in the Xilin Gol League prairie in Inner Mongolia Autonomous Region, and horses were euthanized in October and November 2017. All horses came from the same pasture fence, maintained in same grazing condition, and were fed by same pasture. The dry matter intake (DMI) horse is 16.51 kg day^−1^ per Mongolian (Table A1) (Wei et al., 2015). The animals were examined by a veterinarian to confirm there were no obvious metabolic and gastrointestinal disorders. After euthanasia and dissection, all organs of the gastrointestinal tract were collected by tying up the narrow interface between each segment with ropes, the middle of each segment was collected when the organs were placed horizontally. To ensure the consistency, samples were collected at the same position of each segment. The sampling was as follows: stomach (the pylorus), jejunum (the site 10 cm after the duodenojejunal junction), ileum (the site 10 cm before the ileum–cecum orifice), cecum (the tip of the cecum), ventral colon (the middle of the ventral colon), and dorsal colon (the middle of the dorsal colon; Liu et al., 2019). The contents were stored in a 50‐ml sterile and enzyme‐free centrifuge tube, mixed, and immediately placed in liquid nitrogen, and then cryopreserved at −80°C. The animal experiments were approved by the Animal Welfare Committee of Inner Mongolia Agricultural University, and all procedures were conducted in accordance with the guidelines of the China Animal Protection Association. The characteristics of the individual horses, including age, sex, weight, height, length, bust, hair, and color, are summarized in Table 1.

**Table 1:** Details of the horses used for the characterization of the microbiota present in different compartments of the GIT

Sample	Age	Sex	Weight (kg)	High (cm)	Length (cm)	Bust (cm)	Color	Condition	Reason for euthanasia	Feeding
Horse 1	3	F	275	126	133	145	Black	WNL	Neurological	Grass
Horse 2	3	M	296	135	140	153	Black	WNL	Old wound	Grass
Horse 3	5	M	298	132	140	156	Bay	WNL	Navicular disease	Grass
Horse 4	5	F	285	130	138	153	Gray	WNL	Osteoarthritis	Grass
Horse 5	6	M	310	137	142	156	Chestnut	WNL	Old wound	Grass

Abbreviation: GIT, gastrointestinal tract; WNL, within normal limits.

### 2.2 DNA extraction, 16S rRNA gene PCR, and sequencing

Total genomic DNA was extracted from the GIT samples using the CTAB/SDS method, and the concentration and purity were evaluated by electrophoresing in 1% agarose gels. The distinct regions of the 16S rRNA (V3‐V4 hypervariable regions) were amplified using barcode‐tagged specific primers (16SRNA V3‐V4: 341F‐806R). Each PCR mixture consisted of 15 μl Phusion® High‐Fidelity PCR Master Mix (New England Biolabs), 0.2 μM forward and reverse primers, and ~10 ng template DNA (1 ng/µl) for a final volume of 30 µl. The PCR mixture was denatured at 98°C for 1 min firstly, then followed by 30 cycles of denaturation at 98°C for 10 s, annealing at 50°C for 30 s, and elongation at 72°C for 30 s, and the final elongation was performed at 72°C for 5 min. The PCR products were electrophoresed on a 2% agarose gel and purified by Gene JETTM Gel Extraction Kit (Thermo Scientific).

### 2.3 Library preparation and sequencing

Library construction and sequencing were performed by the Novogene Company. Sequencing libraries were generated using Ion Plus Fragment Library Kit (48 reactions, Thermo Scientific) according to the manufacturer's instructions. The library quality was assessed on the Qubit^®^ 2.0 Fluorometer (Thermo Scientific) and sequenced on an Ion S5 TM XL platform. 400‐bp/600‐bp single‐end reads were generated by sequencing finally.

### 2.4 Data analysis

Single‐end reads were assigned to samples based on their unique barcode and truncated by excising the barcode and primer sequences. The raw reads were first filtered according to the Cutadapt (V1.9.1, http://cutadapt.readthedocs.io/en/stable/) quality control process to obtain high‐quality reads. The latter were compared with the reference database using the UCHIME algorithm (http://www.drive5.com/usearch/manual/uchime/algo.html) (Edgar, Haas, Clemente, Quince, & Knight, 2011) to detect chimaera sequences, which were then removed (Haas et al., 2011). Then, the clean reads were obtained (Table A2). Sequence analyses were performed with Uparse software (v7.0.1001, http://drive5.com/uparse/) (Edgar, 2013), and sequences with ≥97% similarity were assigned to the same operational taxonomic units (OTUs). Representative sequences of each OTU were subjected to species annotation (threshold set at 0.8 to 1) and abundance analysis using the Mothur software and SSU rRNA SILVA128 (http://www.arb-silva.de/) (Accessed Date: November 2017) database (Wang, Garrity, Tiedje, & Cole, 2007) (Quast et al., 2013). With the minimum amount of data in the sample as the standard, the data of each sample were homogenized for subsequent alpha and beta diversity analyses.

To calculate alpha diversity, the OTU table was rarefied and two metrics were calculated, observed species and Shannon index, the observed species is to estimate the amount of unique OTUs found in each sample. Rarefaction curves were generated based on these two metrics. For beta diversity analysis, UniFrac distance was calculated, and unweighted pair group method with arithmetic (UPGMA) mean sample clustering trees were constructed using QIIME software (version 1.9.1). The unweighted UniFrac was used for principal coordinate analysis (PCoA). PCoA can be used for determining principal coordinates and visualizing complex, multidimensional data. Differences in community structure among groups were tested by analysis of molecular variance (AMOVA), and species differences among groups were analyzed with LDA effect size (LEfSe, LDA score of 4). The functional composition of the microorganisms was predicted by the PICRUSt (version 1.1.2) programs. Default parameters were used for all analyses except those specific parameters.

All data analyses were performed using SPSS software, version 22.0. The different parameters of horse GIT were expressed as mean ± standard deviation. Statistical significance was analyzed with ANOVA, and multiple groups were compared using the LSD test.

## 3 RESULTS

### 3.1 Species richness and diversity across the GIT segments

**Figure 1:**
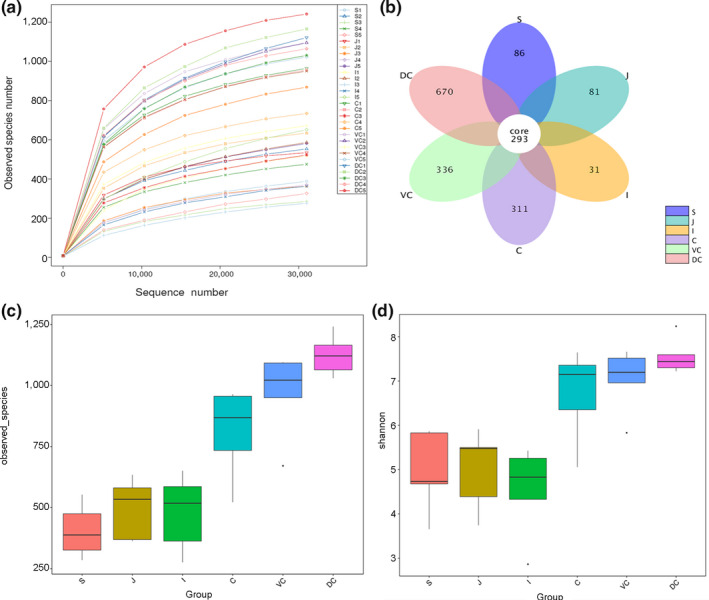
The richness of the luminal Mongolian horse gut microbiota. Rarefaction curves representing the number of phylotypes obtained after sequencing and subsampling 10,100 reads per sample of intestinal content from five horses (a); Venn diagram of OTUs in the luminal Mongolian horse gut microbiota (b); The observed species index intergroup difference box diagram (c); The Shannon index intergroup difference box diagram (d). C, cecum, DC, dorsal colon, I, ileum, J, jejunum, S, stomach, and VC, ventral colon

**Figure A1:**
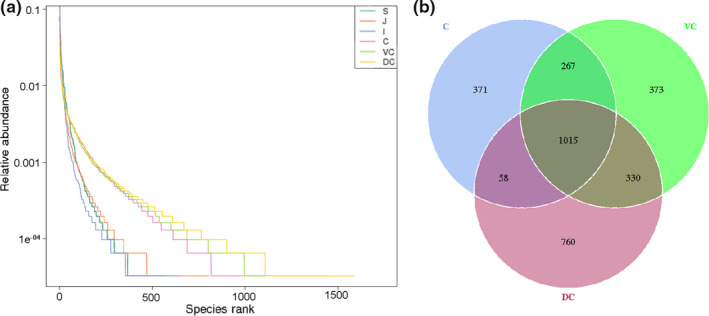
The richness of lower GIT microbiota in the luminal Mongolian horse. Rank abundance curves (a); Venn diagram of OTUs in the luminal Mongolian horse lower GIT microbiota (b)

A total of 2,295,386 valid sequences were obtained, 1,355,813 of which were annotated corresponding to 24,602 OTUs. At the OTU level, all samples of different segments were sequenced approximately to the plateau (Figure 1a), which reflected the richness of species indirectly. The richness was decreased in the following order: dorsal colon (DC) > ventral colon (VC) > cecum (C) > jejunum (J) ≥ ileum (I) > stomach (S). Based on the microbial diversity, the GI segments were stratified in the lower gut (LG) (cecum, ventral colon, dorsal colon) and the upper gut (UG) (stomach, jejunum, ileum), with greater richness seen in the LG (Figure A1‐A). OTU cluster analysis indicated that a total of 293 OTUs were in different GIT segments, which could be divided into 10 phyla in the GIT (Figure 1b) and 16 phyla in the LG (Figure A1‐B), the result indicated that there was a greater richness in the LG. The Venn diagram of the LG indicated that the proportion of specific OTUs in the cecum, ventral colon, and dorsal colon were 11.69%, 11.79%, and 24.95%, respectively. The proportion of common OTUs was 31.98%. The alpha diversity index analysis showed significantly higher microbial diversity in the individual LG segments than different UG segments (*p* < .001; Figure 1c,d), whereas no significant differences were observed among the individual segments of the LG or those of the UG.

### 3.2 Microbial abundance and composition in horse GIT

**Figure 2:**
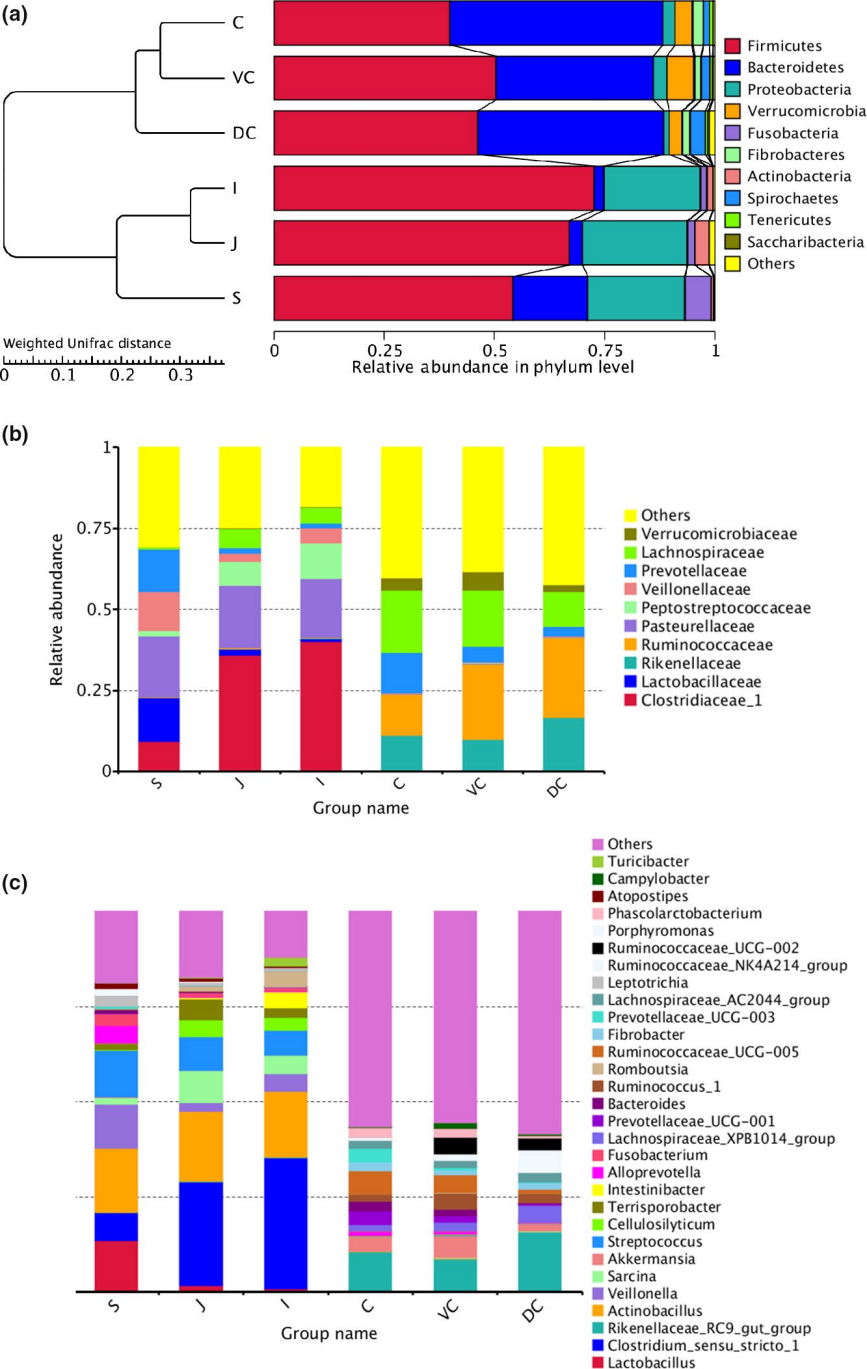
The relative abundance of luminal Mongolian horse GIT microbiota. UPGMA clustering analysis with weighted UniFrac distance matrix on the left and relative abundance of bacteria on the right in each group at the phylum (a), family (b), and genus levels (c)

**Figure A2:**
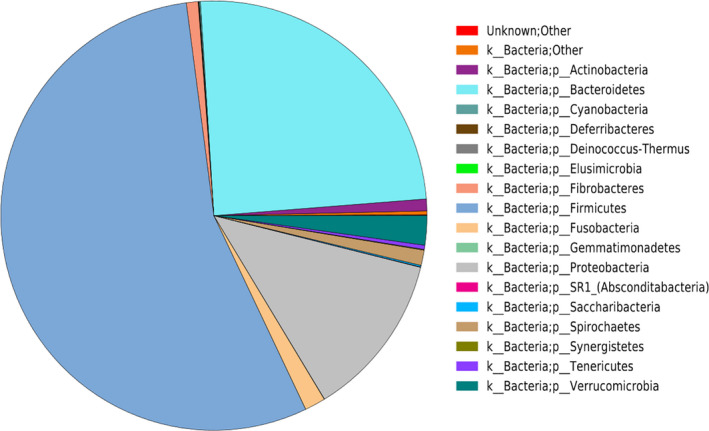
Distribution of the GIT microbiota composition

The OTU sequences of the entire horse GIT were classified into 26 phyla, and the phyla with greatest abundances were the Firmicutes (55.01%), Bacteroidetes (24.76%), and Proteobacteria (12.43%) (Figure A2). However, there were significant differences in the abundances of Firmicutes, Spirochetes (*p* < .05), Bacteroidetes, Verrucomicrobia, Fibrobacteres (*p* < .01), Proteobacteria, and Tenericutes (*p* < .001) between the UG and LG (Figure 2a; Table A3). While the thick‐walled Firmicutes was the most abundant phylum in the UG, the relative abundance of Firmicutes and Bacteroides was similar in the LG (Table A4). The results of analysis by individual segments showed Firmicutes were significantly more abundant in the mid‐ileum than stomach (*p* = .039), cecum (*p* < .001), ventral colon (*p* = .015), and dorsal colon (*p* = .005). Firmicutes were also more abundant in the jejunum than cecum (*p* = .004) and DC (*p* = .022). Bacteroidetes was more abundant in the cecum than the stomach (*p* < .001), jejunum (*p* < .001), ileum (*p* < .001), and ventral colon (*p* = .02), and also in the dorsal colon than the stomach, jejunum, and ileum (*p* < .001 for all). Proteobacteria was more abundant in the jejunum, stomach, and ileum than the cecum, ventral colon, and dorsal colon (*p* < .001, *p* = .001, and *p* < .001, respectively). The abundance of Verrucomicrobia was greater in the ventral colon than the stomach (*p* = .03), jejunum (*p* = .031), and ileum (*p* = .031), and that of Fusobacteria was greater in the stomach than the jejunum (*p* = .009), ileum (*p* = .007), cecum (*p* = .001), ventral colon (*p* = .001), and dorsal colon (*p* < .001). Actinobacteria was more abundant in the jejunum than the stomach (*p* = .03), cecum (*p* = .012), ventral colon (*p* = .012), and dorsal colon (*p* = .011). Spirochetes was more abundant in the dorsal colon than the stomach, jejunum, ileum, cecum (*p* < .001 for all), and ventral colon (*p* = .002), whereas Tenericutes was more abundant in the cecum and ventral colon than the stomach, jejunum, and ileum (*p* < .001 for all).

**Figure 3:**
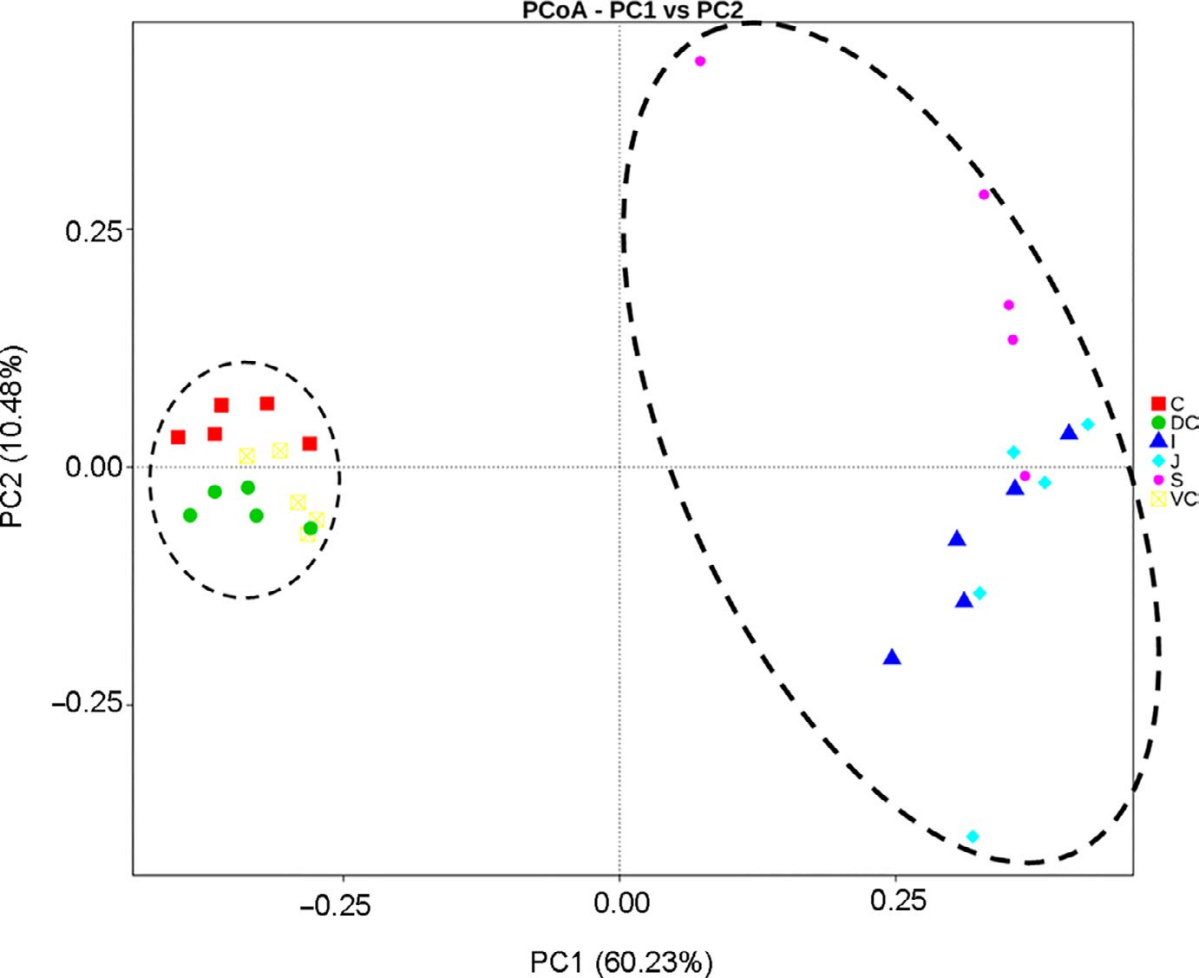
Principal coordinate analysis (PCoA) with clustering representing the dissimilarity of bacterial structure found among samples from Mongolian horse GIT compartments. C, cecum, DC, dorsal colon, I, ileum, J, jejunum, S, stomach, and VC, ventral colon

**Figure 4:**
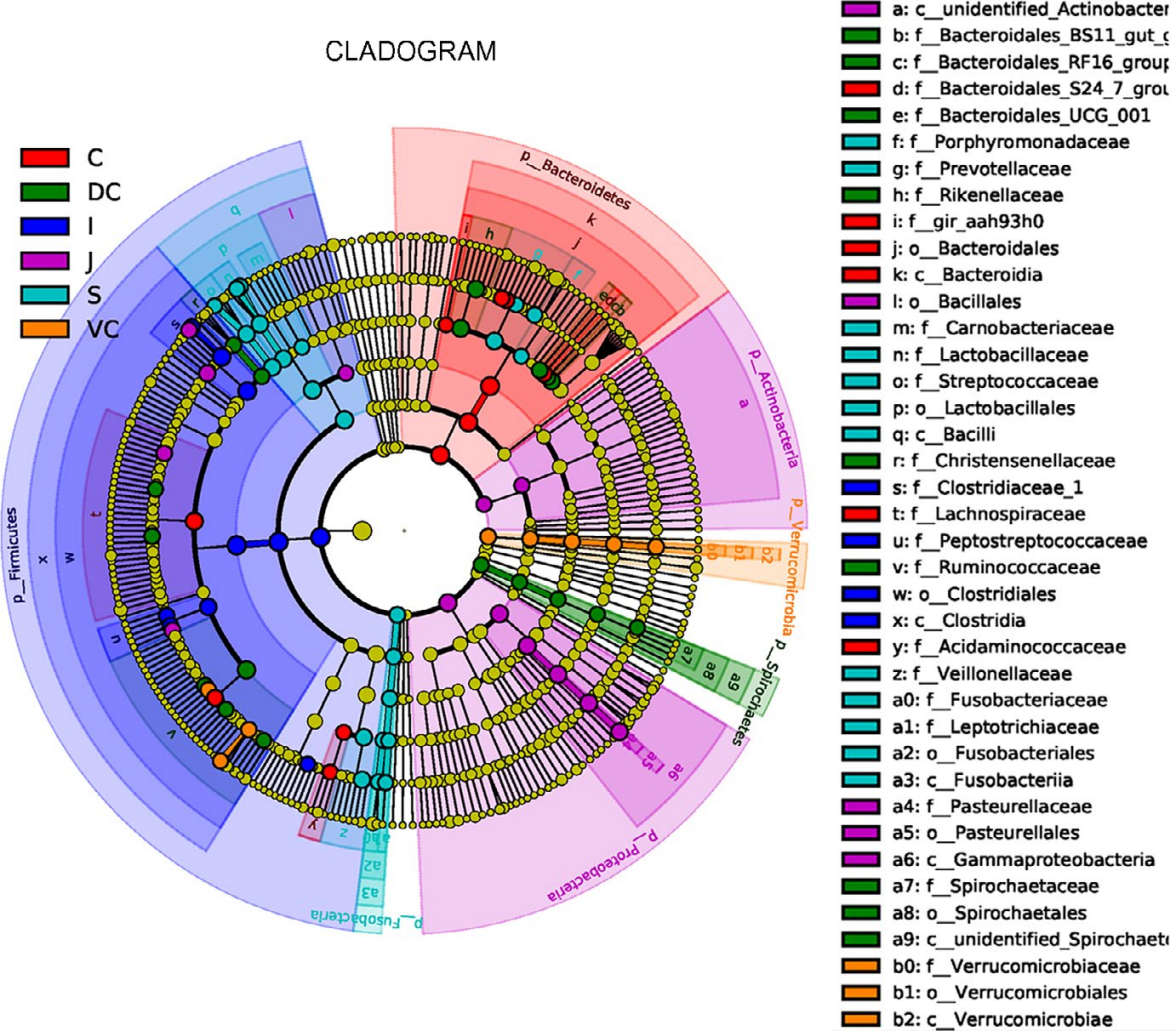
Taxonomic CLADOGRAM reporting the different taxon abundances among GIT groups. C, cecum, DC, dorsal colon, I, ileum, J, jejunum, S, stomach, and VC, ventral colon

**Figure A3:**
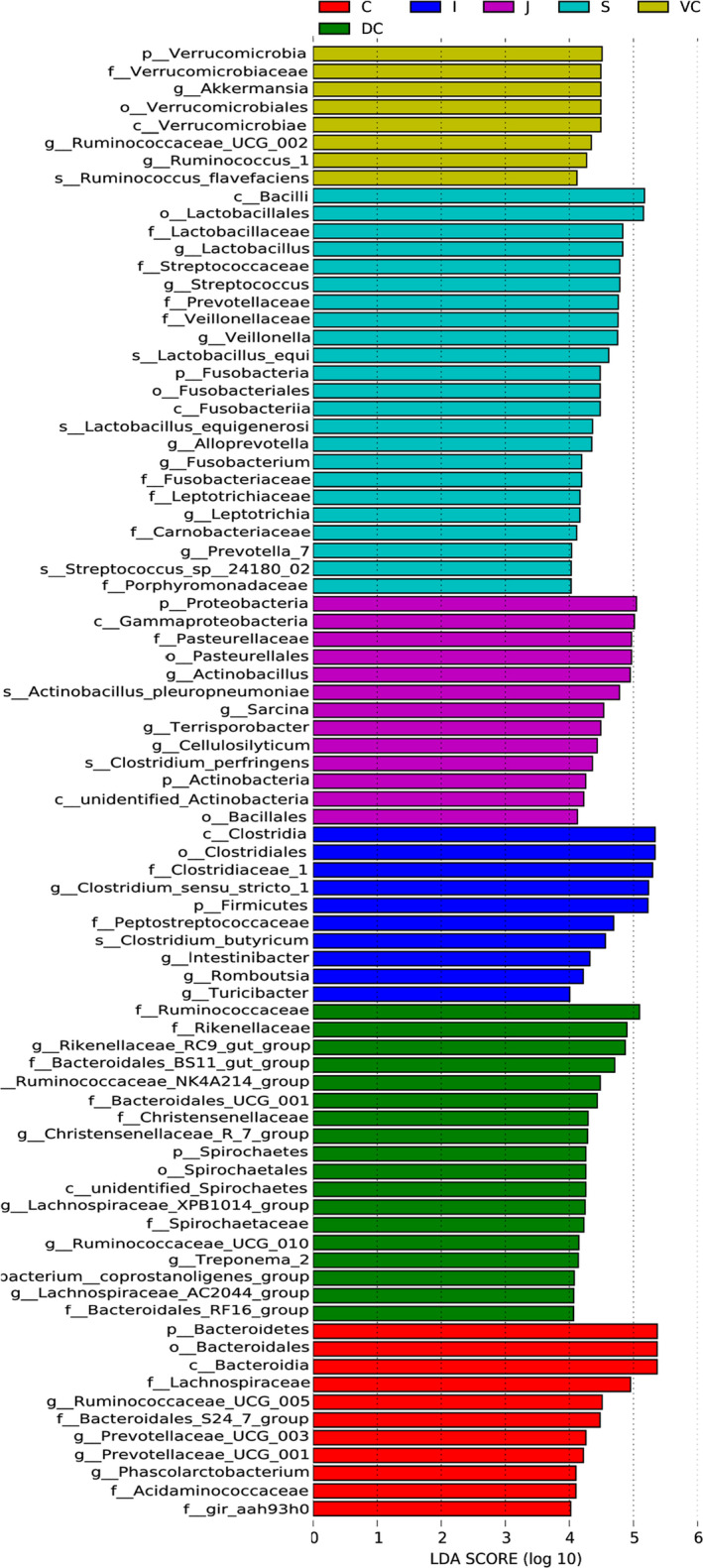
Histogram of the LDA scores calculated for differentially abundant features at the genus level among GIT groups (only the genera LDA scores above 4 are shown). C, cecum, DC, dorsal colon, I, ileum, J, jejunum, S, stomach, and VC, ventral colon

At the genus level, significant differences were also seen between the microbial compositions of the small and large intestines, whereas those of the cecum and colon were more consistent (Figure 2c). The abundance of all genus did not exceed 35% in the UG, and only slight differences were seen between the abundance of different genera in the LG. However, the relative abundance of microorganisms across the different GIT segments was significantly different (Table A5). The results of AMOVA showed that the microbial community structures were significantly different across the distinct GIT regions (*p* < .05; *F* = 12.26), while those of the jejunum, ileum, cecum, and VC were similar (Table 2). To assess the structural differences between samples better, all OTUs were subjected to PCoA based on the weighted UniFrac distance (Figure 3). The samples were formed into two distinct clusters, representing UG and LG, along with the main component 1 (PC1, contribution value of 60.23%) (Figure 3). The UG samples were more dispersed, indicating that there was a greater difference in microbial communities across the segments. In contrast, the LG samples were clustered relatively, indicating higher compositional similarity. The UPGMA clustering tree also showed distinct microbial microbiota in the different parts of the GIT, with those of the UG and LG forming two branches (Figure 2). The linear discriminant analysis (LDA) effect size (LEfSe) values were used to determine the taxonomic biomarkers between GIT segments (Costa et al., 2012), the result revealed 28 microorganisms with different biological relevance across the segments (Figure 4) and 83 microorganisms with LDA values greater than 4 (Figure A3). To summarize, the abundance of Fusobacteria, Proteobacteria and Actinobacteria, Firmicutes, Bacteroidetes, Verrucomicrobia, and Spirochetes was the highest in the stomach, jejunum, ileum, cecum, ventral colon, and dorsal colon, respectively.

**Table 2:** Analysis of molecular variance among luminal microbiota samples in sequential regions of the equine gastrointestinal tract

	AMOVA					
	S	J	I	C	VC	DC
S		**0.02**	**0.015**	**0.009**	**0.008**	**0.005**
J	2.9876		0.929	**0.008**	**0.006**	**0.005**
I	3.2498	0.3294		**0.006**	**0.007**	**0.011**
C	13.0352	20.1294	24.04		0.190	**0.005**
VC	11.7801	17.2003	20.386	1.5424		**0.018**
DC	16.5085	25.821	33.8992	5.3081	2.7487	

Note

Bonferroni‐corrected *p*‐values of pairwise comparisons are shown in the upper right, with significant differences depicted in bold; *F*‐values are shown in the lower left.

Abbreviations: C, cecum, DC, dorsal colon, I, ileum, J, jejunum, S, stomach, and VC, ventral colon.

### 3.3 Putative functions of the GIT microbiota

**Figure 5:**
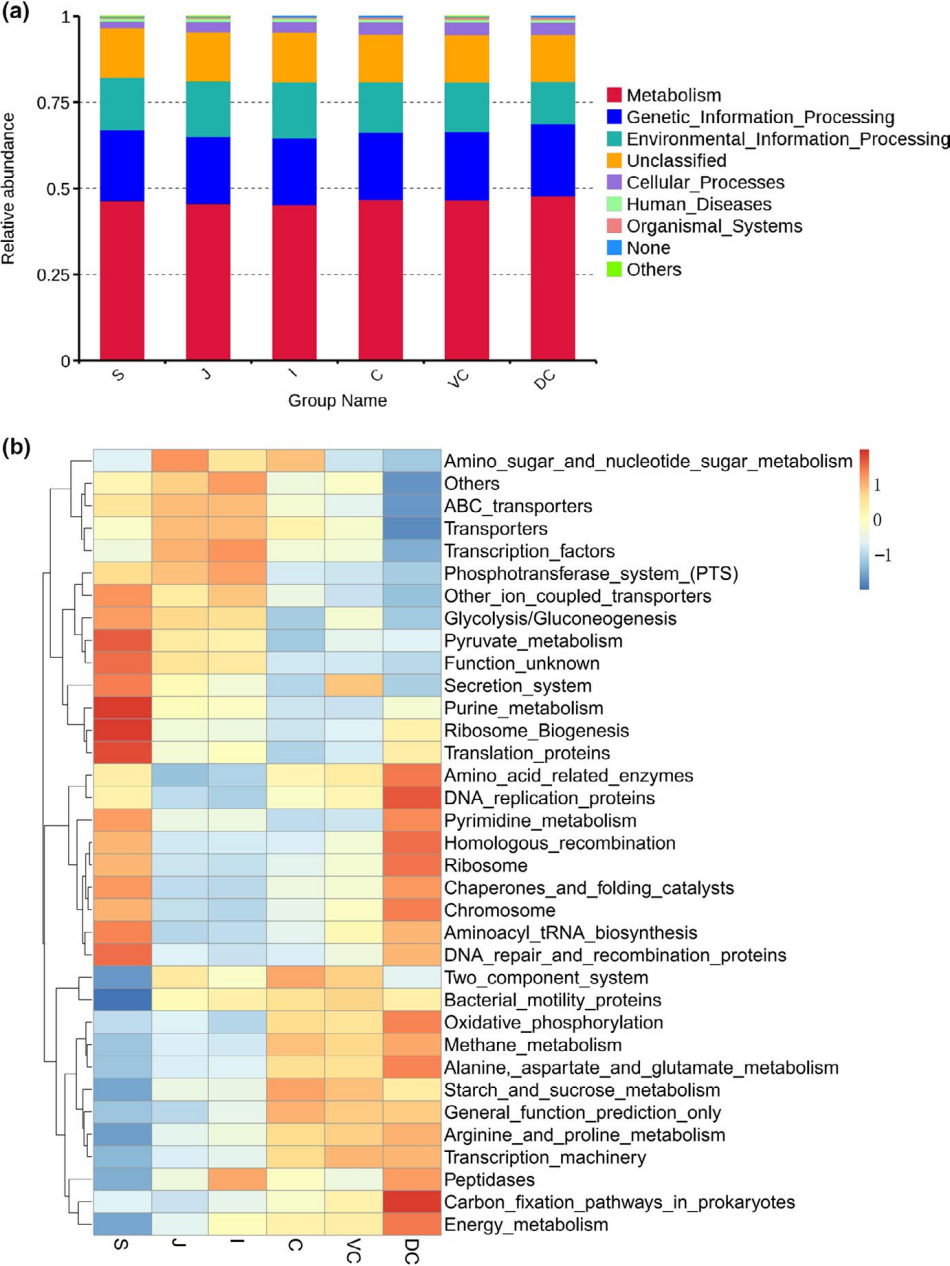
Functional analysis of the bacterial community in the gastrointestinal tract of Mongolian horses. The functional relative abundance histogram is at level 1 (a); heat map of PICRUSt gene predicted function is at level 3 (b). C, cecum, DC, dorsal colon, I, ileum, J, jejunum, S, stomach, and VC, ventral colon

PICRUSt and the KEGG (Kyoto Encyclopedia of Genes and Genomes) database were used to predict the metabolic functions of the GIT microbiota (Figure 5a). The following seven pathways were identified in the primary layer: metabolism (45.32%–47.92%), genetic information processing (19.35%–20.86%), environmental information processing (13.32%–16.29%), unclassified (13.68%–14.41%), cellular processes (1.95%–3.61%), human diseases (0.69%–0.84%), and organic systems (organismal systems, 0.46%–0.77%).

The top 35 predicted functions were screened based on the functional annotation and abundance in the third‐order layer (Figure 5b), and a three‐level functional abundance cluster heat map was drawn. The intensity of the red color indicated abundance. The functional abundance was different across the six segments of the intestine, along with the microbial functions in each part. Eleven functions (including glycolysis/gluconeogenesis) were in the stomach, three functions (including amino sugar and nucleotide sugar metabolism) were in the jejunum, three (including phosphatase and phosphotransferase system) were in the ileum, four (including starch and sucrose metabolism) were in the cecum, bacterial motility proteins were in the ventral colon, and 12 functions of methane metabolism in the dorsal colon were more abundant than in the other segments.

## 4 DISCUSSION

Compared with traditional isolation methods, the next‐generation sequencing appears more efficient to analyzing microbiome structures, especially for the species that are hard to cultivate in vitro (Zhang et al., 2016). Therefore, this technique had been used extensively to characterize the intestinal microbiota of several species (Kim, Gu, Lee, Joh, & Kim, 2012; Orpin, 1981; Wu et al., 2016; Yang et al., 2017; Zhang et al., 2016; Zhou et al., 2016). Present studies on the gut microbiota were focused on fecal samples, which only represent the microbial structures of the right dorsal colon but not the entire gut microbiota. Therefore, direct sampling of the different parts of the GIT can reflect the function of the coevolving bacterial communities in complex mammalian ecosystems (Isaacson & Kim, 2012; Willing et al., 2009) more accurately. At the same time, the study shows that the fecal microbial diversity of wild horses is higher than that of captive horses (Metcalf et al., 2017). Therefore, this paper adopts grazing to simulate the natural state as much as possible.

### 4.1 Composition of the GIT microbiota of the Mongolian horse

The composition of the intestinal microbiota is the result of long‐term evolutionary adaptation of the host to its diet; therefore, there are great differences among herbivores, carnivores, and omnivores. Herbivores have a higher proportion of Firmicutes and Bacteroides, reflecting the high cellulose content from ingested plants (Isaacson & Kim, 2012). In the gut of Mongolian horse, Firmicutes and Bacteroidetes also play the dominant role in the microbiota, accounting for more than 79% of the gut microbes. Studies have shown that these phyla facilitated the digestion and utilization of plant‐derived foods (De Filippo et al., 2010; Xu et al., 2015).

The proportion of Firmicutes and Proteobacteria was the highest in microbiota in the foregut of the Mongolian horses. Proteobacteria maintains the stability of the intestinal microbiota structure and is a key indicator of mammal gut health (Shin, Whon, & Bae, 2015). The Proteobacteria *Actinobacillus* of the family Pasteurellaceae was also abundant in the UG and forms part of the normal microbiota of the anterior intestine of ruminants. However, *Actinobacillus* is a conditional pathogen that can cause diarrhea, meningitis, pneumonia, pyogenic nephritis or septic polyarthritis (snoring or joint disease), and sepsis, indicating that its balance is critical to the health of the animal (Layman, Rezabek, Ramachandran, Love, & Confer, 2014). In the hind or lower gut, Firmicutes and Bacteroidetes were predominant, which demonstrated that the LG is the main region for fermentation of plant fiber.

### 4.2 Diversity of the GIT microbiota in Mongolian horses

As mentioned above, Firmicutes and Bacteroidetes were the dominant bacteria at a ratio of 1:1 in the LG of the horses, the result contradicted the observations using fecal samples (Costa et al., 2012; Costa, Stampfli, et al., 2015; Schoster, Mosing, Jalali, Staempfli, & Weese, 2016; Zhao et al., 2016). However, this result is consistent with studies on microbial communities in different parts of the intestine (Costa, Silva, et al., 2015; Ericsson, Johnson, Lopes, Perry, & Lanter, 2016). Therefore, the feces do not fully represent the entire gut microbiota. In addition, previous studies indicated that the proportion of dominant intestinal microbiota is dependent on the geographical location or seasonal feed (Ericsson et al., 2016), but the availability in Mongolian horses needs further investigation. We observed distinct microbial communities in the different parts of the GIT, but the compositions of adjacent parts were usually similar (except for the ileum and cecum). The greater microbial diversity in the distal gut indicated a more complex microenvironment in that region. This is in agreement with studies that the ecology of the GIT is not static but with significant regional changes (Weese et al., 2015). Based on the gut microbiota, the equine GIT could be divided into two distinct regions: the hindgut region consisting of the cecum, ventral colon, and dorsal colon, and the foregut comprising of the stomach, jejunum, and ileum. While the different parts of the hindgut had similar microbiota, those of the foregut microbes were highly variable among the specific parts, as well as in different horses. As shown in the PCoA plot, individual horses differed most in the stomach or gastric microbiota. This may reflect the higher rate of throughput in the upper GIT, as well as the continuous introduction of environmental bacteria into the pasture.

The stomach mainly harbored the Firmicutes, Proteobacteria, and Bacteroidetes phyla and the *Actinobacillus*, *Lactobacillus*, *Streptococcus,* and *Veillonella* genera, which contradicted the results of Perkins et al. (2012). In addition, the *Fusobacteria*, *Leptotrichia*, and *Alloprevotella* genera were significantly more abundant than the other parts of the intestine. *Fusobacteria* produces VFAs, such as acetic acid, propionic acid, and butyric acid, which are essential for the absorption of electrolytes and the regeneration of mucosal epithelial cells, which are instrumental in preventing inflammation and cancer (Perkins et al., 2012). The jejunum and ileum had similar microbiota composition, possibly due to the proximity or the small sample size. Consistent with the studies by Dougal and Hayashi (Dougal et al., 2012; Hayashi, Takahashi, Nishi, Sakamoto, & Benno, 2005), Proteobacteria and Actinobacteria were the most abundant phyla in the jejunum and predominantly included *Actinobacillus*. Firmicutes was the most abundant phylum (72%) in the mid‐ileum and mainly included the Clostridiaceae (Cymbidaceae) family, the *Clostridium*_sensu_stricto_1 (*C. sinensis*) and the *Turicibacter* genera, all of which were significantly different in the stomach, cecum, VC, and DC. This was inconsistent with the findings of Dougal et al. (2013). The horse ileum also harbored Proteobacteria (22%) and lower Bacteroides (2%), similar to the human ileum (Booijink et al., 2010; Durban et al., 2011). Therefore, the ileal microbiota of Mongolian horses and other mammals appear highly conserved and could be related to the structure and function of the ileum.

Cecum and colon are the major sites of microbial hydrolysis and fermentation to produce VFAs, which are correlated with high abundance of Bacteroidetes and Firmicutes observed in these regions. Dynamic changes in the two phyla are closely related to obesity, and their proportion is an indicator of metabolism (Costa, Stampfli, et al., 2015; Ley, Turnbaugh, Klein, & Gordon, 2006). A high Firmicutes‐to‐Bacteroidetes (FD/BD) ratio is conducive to energy absorption and storage since Firmicutes can ferment more short‐chain fatty acids (SCFAs) to promote fat accumulation (Backhed et al., 2004; Ley et al., 2005). The FD/BD ratio in this population of five Mongolian horse guts was ~0.82, indicating low‐fat deposition, and correlated with the high roughage diet of the horses. Verrucomicrobia and Spirochetes are abundant in the colon (abdominal and dorsal colon), which is consistent with the hindgut microbes of Hokkaido horses, indicating high microbial diversity in both species (Yamano, Koike, Kobayashi, & Hata, 2008). In the LG, the predominant families were Ruminococcaceae (*p* = .203), Lachnospiraceae (*p* = .157), Rikenellaceae (*p* = .122), and Prevo Section (Prevotellaceae, *p* = .068) (Figure 2b). The Ruminococcaceae and Lachnospiraceae families are abundant in the hindgut of many animals, including horses, and are also associated with many intestinal diseases such as inflammatory bowel disease (IBD) (Dougal et al., 2013; Frank et al., 2007). The hindgut microbiota can produce a large amount of butyrate, which affects the health of the colonic mucosa (Brown et al., 2011; Jalanka‐Tuovinen et al., 2011; Pryde, Duncan, Hold, Stewart, & Flint, 2002).

The Ruminococcaceae_UCG‐005, *Phascolarctobacterium*, Prevotellaceae_UCG‐003, *Bacteroides*, and *Fibrobacter* genera were significantly more abundant in the cecum than in the other parts of the GIT, while the relative abundances of Ruminococcus_1, Ruminococcaceae_UCG‐002, *Campylobacter* (*Centida*), and *Akkermansia* (*Ekmania*) genera were the highest in the ventral colon. The Ruminococcaceae_NK4A214_group, Lachnospiraceae_XPB1014_group, Lachnospiraceae_AC2044_group, Rikenellaceae_RC9_gut_group (Reuters), and Prevotellaceae_UCG‐001 (*Prevoella*) genera were abundant in the dorsal colon. *Fibrobacter* (*Bacillus*) and Ruminococcus_1 (Ruminococcus) are cellulose‐degrading bacteria and were abundant in the hindgut, along with *Akkermansia*, which is more abundant in the ventral colon of Mongolian horses (5.7%). This bacterium is an appealing candidate to become a human probiotic because of negative correlation with the incidence of obesity, diabetes, inflammation, and metabolic disorders (Everard et al., 2013; Hansen et al., 2012; Png et al., 2010; Wang, Bose, Kim, Han, & Kim, 2015). Only four previous studies (Costa, Stampfli, Allen‐Vercoe, & Weese, 2016; Costa, Stampfli, et al., 2015; Rodriguez et al., 2015; Zhao et al., 2016) described the genus *Akkermansia* in the equine intestinal microbiota, which was only found in stool samples. In this study, for the first time, we found the ventral colon had the highest content of *Akkermansia* in the gastrointestinal tract of Mongolian horses, which supports further study of this bacterium.

### 4.3 Functional prediction of the Mongolian horse intestinal microbiota

In previous studies, there was no prediction of the function of gastrointestinal flora in different parts of the gastrointestinal tract of horses (Costa, Silva, et al., 2015; Ericsson et al., 2016). This study predicted the functions of the bacterial communities for the first time. In terms of functional diversity, the gut microbiota of the Mongolian horse was enriched in seven pathways, with metabolism, genetic information processing, and environmental information processing as the top three functions. Despite the diversity of the microbial species across the different parts of the GIT, the functional abundance was similar, indicating that the core microbial functions may have species specificity in the GIT. The three‐level functional abundance clustering clearly demarcated the anterior and the posterior intestine microbiota, indicating regional specificity in bacterial functions. However, the predictive power of PICRUSt is limited, and a combination of metagenomic sequencing, related functional gene analysis, and metabolomic profiling can elucidate the functions of the gut microbiota more accurately. In addition, the small sample size in our study may reduce the statistical significance of the differences among the different GIT regions, especially that of the stomach, and may have underestimated the complexity of the microbial communities and the intersample fluctuations. Although this is the first systematic study on the microbial population of the entire GIT of Mongolian horses, further research is needed to determine the effects of other factors such as age, geographical location, and seasonal diet. The influences of these factors on horse intestinal microbiota were not yet clear.

The resolution of the 16S rRNA amplicon sequencing used in this study was limited. Compared with whole‐genome sequencing, targeted sequencing of the 16Sr RNA gene pool can only classify microorganisms at the level of species, and most of the sequences are only annotated at the level of family or genus. Although changes were detected in the composition of multiple microbial communities in this technique, some unclassified flora may still be ignored.

Although the materials collected from various parts of the gastrointestinal tract appeared uniform, the analysis results of a small number of samples may not represent the whole gastrointestinal tract. Multiple iterations of techniques to solve these problems are costly and of limited value.

## 5 CONCLUSIONS

The microbial communities of the different parts of the Mongolian horse GIT were significantly different, and there was greater diversity between the LG and UG. Direct sampling of the different segments of GIT provided a more complete diagram of the gut microbiota compared with fecal analysis. The vegetarian diets and adaptability of Mongolian horses were likely related not only to their stable and complicated gastrointestinal microbiota but also to their special herbivorous digestive physiology.

## CONFLICT OF INTEREST

The authors declare no conflict of interest.

## AUTHOR CONTRIBUTIONS

Shaofeng Su: Data curation; Formal analysis; Methodology; Project administration; Software; Validation. Yiping Zhao: Data curation; Formal analysis; Funding acquisition. Zongzheng Liu: Data curation; Funding acquisition; Investigation; Software; Writing‐original draft. Guiqin Liu: Formal analysis; Methodology; Supervision. Ming Du: Funding acquisition; Investigation. Jing Wu: Formal analysis; Methodology. Dongyi Bai: Methodology; Visualization. Bei Li: Data curation; Formal analysis; Investigation; Supervision. Gerelchimeg Bou: Data curation; Methodology. Xinzhuang Zhang: Conceptualization; Data curation; Formal analysis. Manglai Dugarjaviin: Conceptualization; Data curation; Formal analysis; Funding acquisition; Project administration.

## ETHICS STATEMENT

The animal experiments were approved by the Animal Welfare Committee of Inner Mongolia Agricultural University, and all procedures were conducted in accordance with the guidelines of the China Animal Protection Association.

## Data Availability

Data are available from the National Centre for Biotechnology Information Sequence Read Archive repository under the BioProject PRJNA524207 (https://www.ncbi.nlm.nih.gov/bioproject/PRJNA524207).

## APPENDIX A

**Table A1:** Estimation of dry matter intake by grazing pasture Mongolian horses

Treatment	Standing yield		Number of horses grazing (*N*)	Grazing days (D)	Grazing area (H)	The DMI per horse (PD)
	Fresh grass	Hay				
Button cage	230.12	110.10	26	34	35.23	16.51 ± 4.09
Buckle cage	156.61	68.69				

Note

The cage technique was used as follows: ten 1.5 m × 1.5 m grazing cages were placed within 35.23 ha pasture, and after 34‐day grazing of 26 horses, the forage inside the cages and outside the cages in ten random areas was clipped. The weight of fresh forage was measured, and after drying, the daily dry matter intake of each horses was calculated according to the formula.

Equation

$PD=\frac{\left(A_{1}-A_{2}\right)\times H}{D\times N}$

PD: average daily dry matter intake per horse (kg/day); A1: the weight of dry forage inside the cages (g/m^2^); A2: the weight of dry forage outside the cages (g/m^2^); H: grazing area (ha); D: grazing days (d); *N*: number of horses grazing.

**Table A2:** Data preprocessing statistics and quality control

Sample name	Raw reads	Clean reads	AvgLen	Q20	GC (%)	Effective (%)
S1	90,585	73,788	425	81.63	51.61	81.46
S2	98,791	82,327	421	83.21	51.82	83.33
S3	68,040	56,342	427	81.88	50.79	82.81
S4	82,014	70,854	422	82.14	51.30	86.39
S5	96,887	79,844	423	83.20	52.33	82.41
J1	61,172	51,050	412	82.45	53.03	83.45
J2	59,552	50,242	417	82.89	52.21	84.37
J3	61,896	55,809	413	84.32	52.39	90.17
J4	66,417	57,663	417	81.50	52.10	86.82
J5	50,270	42,694	422	80.87	51.85	84.93
I1	95,415	81,279	409	84.18	52.65	85.18
I2	85,419	73,445	415	84.27	52.66	85.98
I3	88,472	74,295	413	84.03	52.54	83.98
I4	68,976	56,355	416	83.41	52.16	81.70
I5	88,063	73,236	422	83.08	51.91	83.16
C1	99,816	91,416	417	82.07	52.28	91.58
C2	93,470	85,400	417	83.17	51.90	91.37
C3	99,126	87,429	417	82.90	50.75	88.20
C4	96,382	87,077	415	83.34	52.31	90.35
C5	98,692	91,563	417	83.61	52.16	92.78
VC1	89,719	80,405	415	83.48	52.59	89.62
VC2	104,706	93,704	414	84.27	52.67	89.49
VC3	85,569	74,979	415	83.68	51.64	87.62
VC4	96,420	83,924	414	83.87	52.60	87.04
VC5	93,895	86,179	416	84.48	52.02	91.78
DC1	103,526	99,032	417	83.12	53.00	95.66
DC2	101,768	95,426	416	83.26	52.57	93.77
DC3	94,374	86,551	415	82.11	52.78	91.71
DC4	91,961	88,045	417	82.33	52.89	95.74
DC5	90,325	85,003	415	82.84	53.17	94.11

Note

Raw reads: filter out the sequences of low‐quality bases; clean reads: After filtering the chimera, the final sequence is used for subsequent analysis; AvgLen: average length of clean reads; Q20: the percentage of bases whose mass value is greater than 20 in clean reads; GC (%): GC base content in clean reads; effective (%): the number of clean reads versus the number of raw reads.

Abbreviations: C, cecum, DC, dorsal colon, I, ileum, J, jejunum, S, stomach, and VC, ventral colon.

**Table A3:** Comparisons of the relative abundances of LG and UG microbiota at the phylum level

Phylum	Relative abundance^a^	The upper GIT^b^	The lower GIT^c^
*Firmicutes*	0.5501	0.6455 ± 0.0944*	0.4547 ± 0.0533
*Bacteroidetes*	0.2476	0.0744 ± 0.0830	0.4208 ± 0.0637**
*Proteobacteria*	0.1243	0.2255 ± 0.0109***	0.0232 ± 0.0103
*Verrucomicrobia*	0.0220	0.0006 ± 0.0003	0.0434 ± 0.0155**
*Fusobacteria*	0.0158	0.0300 ± 0.0262	0.0016 ± 0.0010
*Spirochetes*	0.0115	0.0002 ± 0.00004	0.0229 ± 0.0108*
*Fibrobacteres*	0.0089	0.0002 ± 0.00004	0.0175 ± 0.0043**
*Actinobacteria*	0.0089	0.01670 ± 0.0130	0.0012 ± 0.0002
*Tenericutes*	0.0031	0.0001 ± 0.00002	0.0061 ± 0.0008***
*Saccharibacteria*	0.0012	0.0010 ± 0.0005	0.0013 ± 0.0021

Abbreviations: GIT, gastrointestinal tract; LG, lower gut; IG, upper gut.

^a^ The relative abundance of different flora in the whole gastrointestinal tract at the phylum level.

^b^ The average relative abundance of different flora in different parts of the upper GIT (stomach, jejunum, and ileum) at the phylum level.

^c^ The average relative abundance of different bacterial communities in the lower GIT in different parts (cecum, ventral colon, and dorsal colon) at the phylum level.

*** *p* < .001, ***p* < .01, and **p* < .05.

**Table A4:** Bacterial group comparisons for the relative abundance of GIT microbiota at the phylum level

Phylum	Stomach	Jejunum	Ileum	Cecum	Ventral colon	Dorsal colon
*Firmicutes*	0.5416 ± 0.2548 ABab	0.6687 ± 0.1109 ABab	0.7261 ± 0.1225 Aa	0.3980 ± 0.0516 Bb	0.5040 ± 0.0900 ABab	0.4620 ± 0.0677 ABb
*Bacteroidetes*	0.1701 ± 0.1551 Bb	0.0311 ± 0.0181 Bbc	0.0220 ± 0.0134 Bc	0.4838 ± 0.0819 Aa	0.3564 ± 0.0682 Aa	0.4222 ± 0.0598 Aa
*Proteobacteria*	0.2195 ± 0.07142 Aa	0.2380 ± 0.1198 Aa	0.2190 ± 0.1365 Aa	0.0271 ± 0.0099 Bb	0.0310 ± 0.03341 Bb	0.0116 ± 0.00601 Bb
*Verrucomicrobia*	0.0003 ± 0.0002	0.0009 ± 0.0007	0.0007 ± 0.0004	0.0396 ± 0.0243	0.0604 ± 0.0967	0.0302 ± 0.0140
*Fusobacteria*	0.0601 ± 0.0585 Ab	0.0159 ± 0.0097 ABac	0.0138 ± 0.0103 ABac	0.0018 ± 0.0030 Bc	0.0025 ± 0.0048 Bc	0.0005 ± 0.0005 Bc
*Fibrobacteres*	0.0001 ± 0.0001	0.0002 ± 0.0002	0.0002 ± 0.0001	0.0222 ± 0.0385	0.0138 ± 0.0108	0.0166 ± 0.0133
*Actinobacteria*	0.0056 ± 0.0090	0.0310 ± 0.0367	0.0134 ± 0.0195	0.0014 ± 0.0009	0.0012 ± 0.0005	0.0009 ± 0.0005
*Spirochetes*	0.0001 ± 0.00004 Cc	0.0002 ± 0.00009 Cc	0.0002 ± 0.0002 Cc	0.0144 ± 0.0081 BCb	0.0193 ± 0.0103 ABb	0.0350 ± 0.0114 Aa
*Tenericutes*	0.00009 ± 0.00005 Bb	0.0001 ± 0.0001 Bb	0.00007 ± 0.00005 Bb	0.0068 ± 0.0031 Aa	0.0063 ± 0.0043 Aa	0.0053 ± 0.0036 ABa
*Saccharibacteria*	0.0006 ± 0.0010	0.0016 ± 0.0020	0.0007 ± 0.0012	0.00007 ± 0.0001	0.0001 ± 0.0001	0.0036 ± 0.0033

Note

Capital letters indicate *p* < .001; lowercase letters indicate *p* < .01 (Student's *t* test).

**Table A5:** Bacterial group comparisons for the relative abundance of GIT microbiota at the genus level

Phylum	Family	Genus	Stomach	Jejunum	Ileum	Cecum	Ventral colon	Dorsal colon
*Firmicutes*	*Lactobacillaceae*	*Lactobacillus*	0.1347 ± 0.2326	0.0175 ± 0.0209	0.0091 ± 0.0130	0.0004 ± 0.0002	0.0003 ± 0.0001	0.0004 ± 0.0003
	*Clostridiaceae*	*Clostridium_sensu_stricto_1*	0.0741 ± 0.0740 Bb	0.2724 ± 0.090 Aa	0.3442 ± 0.1032 Aa	0.0031 ± 0.0008 Bb	0.0034 ± 0.0005 Bb	0.0027 ± 0.0005 Bb
		*Sarcina*	0.0182 ± 0.0236	0.0836 ± 0.0912	0.0499 ± 0.0392	0.0007 ± 0.0002	0.0008 ± 0.0002	0.0005 ± 0.0001
	*Veillonellaceae*	*Veillonella*	0.1160 ± 0.1240 a	0.0234 ± 0.0180 ab	0.0458 ± 0.0558 ab	0.0003 ± 0.0001 b	0.0004 ± 0.0001 b	0.0004 ± 0.0001 b
	*Streptococcaceae*	*Streptococcus*	0.1239 ± 0.0714 Aa	0.0872 ± 0.0679 ABa	0.0642 ± 0.0561 ABab	0.0011 ± 0.0004 Bb	0.0009 ± 0.0003 Bb	0.0008 ± 0.0001 Bb
	*Peptostreptococcaceae*	*Terrisporobacter*	0.0169 ± 0.0315	0.0555 ± 0.0722	0.0259 ± 0.0172	0.0006 ± 0.0003	0.0007 ± 0.0001	0.0005 ± 0.0001
		*Intestinibacter*	0.0003 ± 0.0001	0.0033 ± 0.0023	0.0424 ± 0.0767	0.0002 ± 0.0002	0.0004 ± 0.0002	0.0002 ± 0.0001
		*Romboutsia*	0.0003 ± 0.0001 b	0.0139 ± 0.0237 ab	0.0407 ± 0.0428 a	0.0002 ± 0.0001 b	0.0002 ± 0.0001 b	0.0002 ± 0.0001 b
	*Ruminococcaceae*	*Ruminococcaceae_UCG‐005*	0.0002 ± 0.0001 Bb	0.0007 ± 0.0007 Bb	0.0005 ± 0.0003 Bb	0.0609 ± 0.0229 Aa	0.0469 ± 0.0196 Aa	0.0121 ± 0.0022 Bb
		*Ruminococcus_1*	0.0005 ± 0.0005 b	0.0005 ± 0.0002 b	0.0005 ± 0.0002 b	0.0201 ± 0.0127 ab	0.0438 ± 0.0463 a	0.0255 ± 0.0234 ab
		*Ruminococcaceae_NK4A214_group*	0.0001 ± 0.0001 Bc	0.0006 ± 0.0005 Bc	0.0004 ± 0.0002 Bc	0.0077 ± 0.0059 Bbc	0.0176 ± 0.0095 Bb	0.0590 ± 0.0167 Aa
		*Ruminococcaceae_UCG‐002*	0.0002 ± 0.0001 Bb	0.0003 ± 0.0001 Bb	0.0002 ± 0.0001 Bb	0.0009 ± 0.0004 Bb	0.0433 ± 0.0271 Aa	0.0307 ± 0.0164 Aa
	*Lachnospiraceae*	*Cellulosilyticum*	0.0006 ± 0.0004	0.0445 ± 0.0765	0.0339 ± 0.0402	0.0007 ± 0.0006	0.0020 ± 0.0010	0.0006 ± 0.0003
		*Lachnospiraceae_XPB1014_group*	0.0002 ± 0.0001 b	0.0004 ± 0.0002 b	0.0004 ± 0.0002 b	0.0159 ± 0.0115 ab	0.0234 ± 0.0156 ab	0.0432 ± 0.0475 a
		*Lachnospiraceae_AC2044_group*	0.0001 ± 0.0001	0.0005 ± 0.0002	0.0003 ± 0.0001	0.0216 ± 0.0199	0.0182 ± 0.0134	0.0251 ± 0.0313
	*Acidaminococcaceae*	*Phascolarctobacterium*	0.0002 ± 0.0001 b	0.0003 ± 0.0002 b	0.0001 ± 0.00004 b	0.0243 ± 0.0248 a	0.0226 ± 0.0166 ab	0.0072 ± 0.0029 ab
	*Carnobacteriaceae*	*Atopostipes*	0.0139 ± 0.0272	0.0067 ± 0.0098	0.0033 ± 0.0059	0.00003 ± 0.00002	0.00008 ± 0.00002	0.00003 ± 0.00004
	*Erysipelotrichaceae*	*Turicibacter*	0.0001 ± 0.0002 b	0.0012 ± 0.0011 b	0.0222 ± 0.0272 a	0.0001 ± 0.0001 b	0.0001 ± 0.00004 b	0.0001 ± 0.00004 b
*Bacteroidetes*	*Rikenellaceae*	*Rikenellaceae_RC9_gut_group*	0.0007 ± 0.0005 Bb	0.0007 ± 0.0004 Bb	0.0008 ± 0.0005 Bb	0.1018 ± 0.1188 ABab	0.0838 ± 0.0647 ABab	0.1546 ± 0.0426 Aa
	*Prevotellaceae*	*Alloprevotella*	0.0466 ± 0.0557	0.0031 ± 0.0030	0.0023 ± 0.0018	0.0113 ± 0.0056	0.0057 ± 0.0024	0.0017 ± 0.0009
		*Prevotellaceae_UCG‐001*	0.0002 ± 0.0003	0.0001 ± 0.0001	0.0001 ± 0.0002	0.0366 ± 0.0482	0.0172 ± 0.0197	0.0519 ± 0.0035
		*Prevotellaceae_UCG‐003*	0.0065 ± 0.0099 ABb	0.0006 ± 0.0051 Bb	0.0002 ± 0.0002 Bb	0.0360 ± 0.0277 Aa	0.0068 ± 0.0277 ABb	0.0023 ± 0.0013 Bb
	*Bacteroidaceae*	*Bacteroides*	0.0104 ± 0.0137	0.0029 ± 0.0029	0.0009 ± 0.0004	0.0253 ± 0.0499	0.0160 ± 0.0314	0.0011 ± 0.0018
	*Porphyromonadaceae*	*Porphyromonas*	0.0194 ± 0.0305	0.0044 ± 0.0065	0.0003 ± 0.0034	0.0001 ± 0.0001	0.0001 ± 0.0001	0.0002 ± 0.0001
*Proteobacteria*	*Pasteurellaceae*	*Actinobacillus*	0.1684 ± 0.0242 Aa	0.1851 ± 0.1070 Aa	0.1735 ± 0.1133 Aa	0.0020 ± 0.0014 Bb	0.0017 ± 0.0005 Bb	0.0011 ± 0.0003 Bb
	*Campylobacteraceae*	*Campylobacter*	0.0008 ± 0.0009	0.0008 ± 0.0009	0.0006 ± 0.0003	0.0022 ± 0.0028	0.0148 ± 0.0267	0.0037 ± 0.0043
*Verrucomicrobia*	*Akkermansiaceae*	*Akkermansia*	0.0003 ± 0.0001	0.0008 ± 0.0005	0.0006 ± 0.0004	0.0369 ± 0.0223	0.0579 ± 0.0871	0.0202 ± 0.0128
*Fusobacteria*	*Fusobacteriaceae*	*Fusobacterium*	0.0321 ± 0.0532	0.0100 ± 0.0077	0.0084 ± 0.0081	0.0016 ± 0.0028	0.0023 ± 0.0044	0.0004 ± 0.0005
*Fusobacteria*	*Leptotrichiaceae*	*Leptotrichia*	0.0280 ± 0.0341	0.0058 ± 0.0078	0.0054 ± 0.0068	0.0002 ± 0.0001	0.0002 ± 0.0001	0.0001 ± 0.0001
*Fibrobacteres*	*Fibrobacteraceae*	*Fibrobacter*	0.0001 ± 0.0001	0.0002 ± 0.0001	0.0002 ± 0.0001	0.0222 ± 0.0344	0.0138 ± 0.0097	0.0166 ± 0.0119

Note

Capital letters indicate *p* < .001; lowercase letters indicate *p* < .01 (Student's *t* test).

## References

[mbo31020-bib-0001] Argenzio, R. A. (1975). Functions of the equine large intestine and their interrelationship in disease. Cornell Veterinarian, 65(3), 303–330. 237739

[mbo31020-bib-0002] Backhed, F. , Ding, H. , Wang, T. , Hooper, L. V. , Koh, G. Y. , Nagy, A. , … Gordon, J. I. (2004). The gut microbiota as an environmental factor that regulates fat storage. Proceedings of the National Academy of Sciences of the United States of America, 101(44), 15718–15723. 10.1073/pnas.0407076101 15505215 PMC524219

[mbo31020-bib-0003] Booijink, C. C. , El‐Aidy, S. , Rajilic‐Stojanovic, M. , Heilig, H. G. , Troost, F. J. , Smidt, H. , … Zoetendal, E. G. (2010). High temporal and inter‐individual variation detected in the human ileal microbiota. Environmental Microbiology, 12(12), 3213–3227. 10.1111/j.1462-2920.2010.02294.x 20626454

[mbo31020-bib-0004] Brown, C. T. , Davis‐Richardson, A. G. , Giongo, A. , Gano, K. A. , Crabb, D. B. , Mukherjee, N. , … Triplett, E. W. (2011). Gut microbiome metagenomics analysis suggests a functional model for the development of autoimmunity for type 1 diabetes. PLoS One, 6(10), e25792 10.1371/journal.pone.0025792 22043294 PMC3197175

[mbo31020-bib-0007] Costa, M. C. , Arroyo, L. G. , Allen‐Vercoe, E. , Stampfli, H. R. , Kim, P. T. , Sturgeon, A. , & Weese, J. S. (2012). Comparison of the fecal microbiota of healthy horses and horses with colitis by high throughput sequencing of the V3–V5 region of the 16S rRNA Gene. PLoS One, 7(7), e41484 10.1371/journal.pone.0041484 22859989 PMC3409227

[mbo31020-bib-0008] Costa, M. C. , Silva, G. , Ramos, R. V. , Staempfli, H. R. , Arroyo, L. G. , Kim, P. , & Weese, J. S. (2015). Characterization and comparison of the bacterial microbiota in different gastrointestinal tract compartments in horses. The Veterinary Journal, 205(1), 74–80. 10.1016/j.tvjl.2015.03.018 25975855

[mbo31020-bib-0009] Costa, M. C. , Stampfli, H. R. , Allen‐Vercoe, E. , & Weese, J. S. (2016). Development of the faecal microbiota in foals. Equine Veterinary Journal, 48(6), 681–688. 10.1111/evj.12532 26518456

[mbo31020-bib-0010] Costa, M. C. , Stampfli, H. R. , Arroyo, L. G. , Allen‐Vercoe, E. , Gomes, R. G. , & Weese, J. S. (2015). Changes in the equine fecal microbiota associated with the use of systemic antimicrobial drugs. BMC Veterinary Research, 11(1), 19 10.1186/s12917-015-0335-7 25644524 PMC4323147

[mbo31020-bib-0011] De Filippo, C. , Cavalieri, D. , Di Paola, M. , Ramazzotti, M. , Poullet, J. B. , Massart, S. , … Lionetti, P. (2010). Impact of diet in shaping gut microbiota revealed by a comparative study in children from Europe and rural Africa. Proceedings of the National Academy of Sciences of the United States of America, 107(33), 14691–14696. 10.1073/pnas.1005963107 20679230 PMC2930426

[mbo31020-bib-0013] Dougal, K. , de la Fuente, G. , Harris, P. A. , Girdwood, S. E. , Pinloche, E. , & Newbold, C. J. (2013). Identification of a core bacterial community within the large intestine of the horse. PLoS One, 8(10), e77660 10.1371/journal.pone.0077660 24204908 PMC3812009

[mbo31020-bib-0014] Dougal, K. , Harris, P. A. , Edwards, A. , Pachebat, J. A. , Blackmore, T. M. , Worgan, H. J. , & Newbold, C. J. (2012). A comparison of the microbiome and the metabolome of different regions of the equine hindgut. FEMS Microbiology Ecology, 82(3), 642–652. 10.1111/j.1574-6941.2012.01441.x 22757649

[mbo31020-bib-0015] Durban, A. , Abellan, J. J. , Jimenez‐Hernandez, N. , Ponce, M. , Ponce, J. , Sala, T. , … Moya, A. (2011). Assessing gut microbial diversity from feces and rectal mucosa. Microbial Ecology, 61(1), 123–133. 10.1007/s00248-010-9738-y 20734040

[mbo31020-bib-0016] Eckburg, P. B. , Bik, E. M. , Bernstein, C. N. , Purdom, E. , Dethlefsen, L. , Sargent, M. , … Relman, D. A. (2005). Diversity of the human intestinal microbial flora. Science, 308(5728), 1635–1638. 10.1126/science.1110591 15831718 PMC1395357

[mbo31020-bib-0017] Edgar, R. C. (2013). UPARSE: Highly accurate OTU sequences from microbial amplicon reads. Nature Methods, 10(10), 996–998. 10.1038/nmeth.2604 23955772

[mbo31020-bib-0018] Edgar, R. C. , Haas, B. J. , Clemente, J. C. , Quince, C. , & Knight, R. (2011). UCHIME improves sensitivity and speed of chimera detection. Bioinformatics, 27(16), 2194–2200. 10.1093/bioinformatics/btr381 21700674 PMC3150044

[mbo31020-bib-0019] Ericsson, A. C. , Johnson, P. J. , Lopes, M. A. , Perry, S. C. , & Lanter, H. R. (2016). A microbiological map of the healthy equine gastrointestinal tract. PLoS One, 11(11), e0166523 10.1371/journal.pone.0166523 27846295 PMC5112786

[mbo31020-bib-0020] Everard, A. , Belzer, C. , Geurts, L. , Ouwerkerk, J. P. , Druart, C. , Bindels, L. B. , … Cani, P. D. (2013). Cross‐talk between *Akkermansia muciniphila* and intestinal epithelium controls diet‐induced obesity. Proceedings of the National Academy of Sciences of the United States of America, 110(22), 9066–9071. 10.1073/pnas.1219451110 23671105 PMC3670398

[mbo31020-bib-0021] Frank, D. N. , St Amand, A. L. , Feldman, R. A. , Boedeker, E. C. , Harpaz, N. , & Pace, N. R. (2007). Molecular‐phylogenetic characterization of microbial community imbalances in human inflammatory bowel diseases. Proceedings of the National Academy of Sciences of the United States of America, 104(34), 13780–13785. 10.1073/pnas.0706625104 17699621 PMC1959459

[mbo31020-bib-0022] Glinsky, M. J. , Smith, R. M. , Spires, H. R. , & Davis, C. L. (1976). Measurement of volatile fatty acid production rates in the cecum of the pony. Journal of Animal Science, 42(6), 1465–1470. 10.2527/jas1976.4261465x 931822

[mbo31020-bib-0023] Haas, B. J. , Gevers, D. , Earl, A. M. , Feldgarden, M. , Ward, D. V. , Giannoukos, G. , … Birren, B. W. (2011). Chimeric 16S rRNA sequence formation and detection in Sanger and 454‐pyrosequenced PCR amplicons. Genome Research, 21(3), 494–504. 10.1101/gr.112730.110 21212162 PMC3044863

[mbo31020-bib-0024] Hansen, C. H. , Krych, L. , Nielsen, D. S. , Vogensen, F. K. , Hansen, L. H. , Sorensen, S. J. , … Hansen, A. K. (2012). Early life treatment with vancomycin propagates *Akkermansia muciniphila* and reduces diabetes incidence in the NOD mouse. Diabetologia, 55(8), 2285–2294. 10.1007/s00125-012-2564-7 22572803

[mbo31020-bib-0025] Harris, P. A. , Ellis, A. D. , Fradinho, M. J. , Jansson, A. , Julliand, V. , Luthersson, N. , … Vervuert, I. (2017). Review: Feeding conserved forage to horses: recent advances and recommendations. Animal, 11(6), 958–967. 10.1017/S1751731116002469 27881201

[mbo31020-bib-0026] Hayashi, H. , Takahashi, R. , Nishi, T. , Sakamoto, M. , & Benno, Y. (2005). Molecular analysis of jejunal, ileal, caecal and recto‐sigmoidal human colonic microbiota using 16S rRNA gene libraries and terminal restriction fragment length polymorphism. Journal of Medical Microbiology, 54(11), 1093–1101. 10.1099/jmm.0.45935-0 16192442

[mbo31020-bib-0027] Isaacson, R. , & Kim, H. B. (2012). The intestinal microbiome of the pig. Animal Health Research Reviews, 13(1), 100–109. 10.1017/S1466252312000084 22853934

[mbo31020-bib-0028] Jalanka‐Tuovinen, J. , Salonen, A. , Nikkila, J. , Immonen, O. , Kekkonen, R. , Lahti, L. , … de Vos, W. M. (2011). Intestinal microbiota in healthy adults: temporal analysis reveals individual and common core and relation to intestinal symptoms. PLoS One, 6(7), e23035 10.1371/journal.pone.0023035 21829582 PMC3145776

[mbo31020-bib-0029] Kim, K. A. , Gu, W. , Lee, I. A. , Joh, E. H. , & Kim, D. H. (2012). High fat diet‐induced gut microbiota exacerbates inflammation and obesity in mice via the TLR4 signaling pathway. PLoS One, 7(10), e47713 10.1371/journal.pone.0047713 23091640 PMC3473013

[mbo31020-bib-0030] Layman, Q. D. , Rezabek, G. B. , Ramachandran, A. , Love, B. C. , & Confer, A. W. (2014). A retrospective study of equine actinobacillosis cases: 1999–2011. Journal of Veterinary Diagnostic Investigation, 26(3), 365–375. 10.1177/1040638714531766 24742921

[mbo31020-bib-0031] Ley, R. E. , Backhed, F. , Turnbaugh, P. , Lozupone, C. A. , Knight, R. D. , & Gordon, J. I. (2005). Obesity alters gut microbial ecology. Proceedings of the National Academy of Sciences of the United States of America, 102(31), 11070–11075. 10.1073/pnas.0504978102 16033867 PMC1176910

[mbo31020-bib-0032] Ley, R. E. , Turnbaugh, P. J. , Klein, S. , & Gordon, J. I. (2006). Microbial ecology: human gut microbes associated with obesity. Nature, 444(7122), 1022–1023. 10.1038/4441022a 17183309

[mbo31020-bib-0033] Liu, G. Q. , Bou, G. , Su, S. F. , Xing, J. Y. , Qu, H. L. , Zhang, X. Z. , … Dugarjaviin, M. L. (2019). Microbial diversity within the digestive tract contents of Dezhou donkeys. PLoS One, 14(12), e0226186 10.1371/journal.pone.0226186 31834903 PMC6910686

[mbo31020-bib-0034] Metcalf, J. L. , Song, S. J. , Morton, J. T. , Weiss, S. , Orlando, A. S. , Joly, F. , … Orlando, L. (2017). Evaluating the impact of domestication and captivity on the horse gut microbiome. Scientific Reports, 7(1), 15497 10.1038/s41598-017-15375-9 29138485 PMC5686199

[mbo31020-bib-0035] Milinovich, G. J. , Trott, D. J. , Burrell, P. C. , Croser, E. L. , Al Jassim, R. A. M. , Morton, J. M. , … Pollitt, C. C. (2007). Fluorescence in situ hybridization analysis of hindgut bacteria associated with the development of equine laminitis. Environmental Microbiology, 9(8), 2090–2100. 10.1111/j.1462-2920.2007.01327.x 17635552

[mbo31020-bib-0036] Orpin, C. G. (1981). Isolation of cellulolytic phycomycete fungi from the caecum of the horse. Journal of General Microbiology, 123(2), 287–296. 10.1099/00221287-123-2-287 7033458

[mbo31020-bib-0037] Perkins, G. A. , den Bakker, H. C. , Burton, A. J. , Erb, H. N. , McDonough, S. P. , McDonough, P. L. , … Simpson, K. W. (2012). Equine stomachs harbor an abundant and diverse mucosal microbiota. Applied and Environment Microbiology, 78(8), 2522–2532. 10.1128/AEM.06252-11 PMC3318809 22307294

[mbo31020-bib-0038] Png, C. W. , Linden, S. K. , Gilshenan, K. S. , Zoetendal, E. G. , McSweeney, C. S. , Sly, L. I. , … Florin, T. H. (2010). Mucolytic bacteria with increased prevalence in IBD mucosa augment in vitro utilization of mucin by other bacteria. American Journal of Gastroenterology, 105(11), 2420–2428. 10.1038/ajg.2010.281 20648002

[mbo31020-bib-0039] Pryde, S. E. , Duncan, S. H. , Hold, G. L. , Stewart, C. S. , & Flint, H. J. (2002). The microbiology of butyrate formation in the human colon. FEMS Microbiology Letters, 217(2), 133–139. 10.1111/j.1574-6968.2002.tb11467.x 12480096

[mbo31020-bib-0040] Quast, C. , Pruesse, E. , Yilmaz, P. , Gerken, J. , Schweer, T. , Yarza, P. , … Glockner, F. O. (2013). The SILVA ribosomal RNA gene database project: improved data processing and web‐based tools. Nucleic Acids Research, 41(D1), D590–D596. 10.1093/nar/gks1219 23193283 PMC3531112

[mbo31020-bib-0041] Rodriguez, C. , Taminiau, B. , Brevers, B. , Avesani, V. , Van Broeck, J. , Leroux, A. , … Daube, G. (2015). Faecal microbiota characterisation of horses using 16 rdna barcoded pyrosequencing, and carriage rate of clostridium difficile at hospital admission. BMC Microbiology, 15, 181 10.1186/s12866-015-0514-5 26377067 PMC4573688

[mbo31020-bib-0042] Santos, A. S. , Rodrigues, M. A. , Bessa, R. J. , Ferreira, L. M. , & Martin‐Rosset, W. (2011). Understanding the equine cecum‐colon ecosystem: current knowledge and future perspectives. Animal, 5(1), 48–56. 10.1017/S1751731110001588 22440701

[mbo31020-bib-0043] Schoster, A. , Mosing, M. , Jalali, M. , Staempfli, H. R. , & Weese, J. S. (2016). Effects of transport, fasting and anaesthesia on the faecal microbiota of healthy adult horses. Equine Veterinary Journal, 48(5), 595–602. 10.1111/evj.12479 26122549

[mbo31020-bib-0044] Shin, N. R. , Whon, T. W. , & Bae, J. W. (2015). Proteobacteria: microbial signature of dysbiosis in gut microbiota. Trends in Biotechnology, 33(9), 496–503. 10.1016/j.tibtech.2015.06.011 26210164

[mbo31020-bib-0045] Steelman, S. M. , Chowdhary, B. P. , Dowd, S. , Suchodolski, J. , & Janecka, J. E. (2012). Pyrosequencing of 16S rRNA genes in fecal samples reveals high diversity of hindgut microflora in horses and potential links to chronic laminitis. BMC Veterinary Research, 8, 231 10.1186/1746-6148-8-231 23186268 PMC3538718

[mbo31020-bib-0046] Vermorel, M. , & MartinRosset, W. (1997). Concepts, scientific bases, structure and validation of the French horse net energy system (UFC). Livestock Production Science, 47(3), 261–275. 10.1016/S0301-6226(96)01410-8

[mbo31020-bib-0047] Wang, J. H. , Bose, S. , Kim, H. G. , Han, K. S. , & Kim, H. (2015). Fermented Rhizoma Atractylodis Macrocephalae alleviates high fat diet‐induced obesity in association with regulation of intestinal permeability and microbiota in rats. Scientific Reports, 5, 8391 10.1038/srep08391 25684573 PMC4329570

[mbo31020-bib-0048] Wang, Q. , Garrity, G. M. , Tiedje, J. M. , & Cole, J. R. (2007). Naive Bayesian classifier for rapid assignment of rRNA sequences into the new bacterial taxonomy. Applied and Environmental Microbiology, 73(16), 5261–5267. 10.1128/AEM.00062-07 17586664 PMC1950982

[mbo31020-bib-0049] Weese, J. S. , Holcombe, S. J. , Embertson, R. M. , Kurtz, K. A. , Roessner, H. A. , Jalali, M. , & Wismer, S. E. (2015). Changes in the faecal microbiota of mares precede the development of post partum colic. Equine Veterinary Journal, 47(6), 641–649. 10.1111/evj.12361 25257320

[mbo31020-bib-0050] Wei, P. , An, S. Z. , Sun, Z. J. , Li, H. , Deng, H. F. , Zhang, W. G. , … Kasidaer, N. (2015). Preliminary study of dairy Yili horse feed intake under grazing conditions. Pratacultural Science, 32(1), 114–118.

[mbo31020-bib-0051] Willing, B. , Voros, A. , Roos, S. , Jones, C. , Jansson, A. , & Lindberg, J. E. (2009). Changes in faecal bacteria associated with concentrate and forage‐only diets fed to horses in training. Equine Veterinary Journal, 41(9), 908–914. 10.2746/042516409X447806 20383990

[mbo31020-bib-0052] Wu, X. Y. , Zhang, H. H. , Chen, J. , Shang, S. , Wei, Q. G. , Yan, J. K. , & Tu, X. Y. (2016). Comparison of the fecal microbiota of dholes high‐throughput Illumina sequencing of the V3–V4 region of the 16S rRNA gene. Applied Microbiology and Biotechnology, 100(8), 3577–3586. 10.1007/s00253-015-7257-y 26728019

[mbo31020-bib-0053] Xu, B. , Xu, W. , Li, J. , Dai, L. , Xiong, C. , Tang, X. , … Huang, Z. (2015). Metagenomic analysis of the *Rhinopithecus bieti* fecal microbiome reveals a broad diversity of bacterial and glycoside hydrolase profiles related to lignocellulose degradation. BMC Genomics, 16, 174 10.1186/s12864-015-1378-7 25887697 PMC4369366

[mbo31020-bib-0054] Yamano, H. , Koike, S. , Kobayashi, Y. , & Hata, H. (2008). Phylogenetic analysis of hindgut microbiota in Hokkaido native horses compared to light horses. Animal Science Journal, 79(2), 234–242. 10.1111/j.1740-0929.2008.00522.x

[mbo31020-bib-0055] Yang, H. , Huang, X. , Fang, S. , He, M. , Zhao, Y. , Wu, Z. , … Huang, L. (2017). Unraveling the fecal microbiota and metagenomic functional capacity associated with feed efficiency in pigs. Frontiers in Microbiology, 8, 1555 10.3389/fmicb.2017.01555 28861066 PMC5559535

[mbo31020-bib-0056] Zhang, D. , Ji, H. , Liu, H. , Wang, S. , Wang, J. , & Wang, Y. (2016). Changes in the diversity and composition of gut microbiota of weaned piglets after oral administration of Lactobacillus or an antibiotic. Applied Microbiology and Biotechnology, 100(23), 10081–10093. 10.1007/s00253-016-7845-5 27757509

[mbo31020-bib-0057] Zhao, Y. P. , Li, B. , Bai, D. Y. , Huang, J. L. , Shiraigo, W. , Yang, L. H. , … Dugarjaviin, M. (2016). Comparison of fecal microbiota of mongolian and thoroughbred horses by high‐throughput sequencing of the V4 region of the 16S rRNA gene. Asian‐Australasian Journal of Animal Sciences, 29(9), 1345–1352. 10.5713/ajas.15.0587 26954132 PMC5003997

[mbo31020-bib-0058] Zhou, X. Y. , Jiang, X. S. , Yang, C. W. , Ma, B. C. , Lei, C. W. , Xu, C. W. , … Wang, H. (2016). Cecal microbiota of Tibetan Chickens from five geographic regions were determined by 16S rRNA sequencing. Microbiologyopen, 5(5), 753–762. 10.1002/mbo3.367 27139888 PMC5061713

